# Development of a New Adjustable Compression Garment (McBoooon) Made of Non-Stretch Self-Adhesive Fabric

**DOI:** 10.3400/avd.oa.23-00107

**Published:** 2024-04-18

**Authors:** Yasushi Shiraishi, Naomi Kinoto, Atsuko Miyoshi, Kyoko Ishii, Mika Ogawa

**Affiliations:** 1 Shiraishi Cardiovascular Clinic, Takamatsu, Kagawa, Japan

**Keywords:** adjustable compression garment, self-sticking, inelastic, stiffness, adherence

## Abstract

**Objectives:** To assess the physical features and compression characteristics of a newly developed adjustable compression garment, McBoooon (Mc).

**Methods:** Twelve healthy volunteers were recruited to assess the compression characteristics. The interface pressure (IP) was continuously measured to calculate the static (SSI) and dynamic stiffness indices (DSI). Additionally, the peak flow velocity (PV) of the popliteal vein during ankle dorsiflexion was measured using ultrasonography. Each parameter was compared between ASHIKA stockings (AS), Mc applied at the same resting pressure as AS (Mc1), and Mc applied at a resting pressure approximately twice that of Mc1 (Mc2).

**Results:** SSI and DSI were significantly different, increasing in the order AS < Mc1 < Mc2 (p <0.01). Although the PV was significantly higher in the compression group than in the control group (p <0.05), no significant differences were found among the three groups.

**Conclusion:** The physical features and compression characteristics of Mc were clarified. The high stiffness of this garment improves the adherence to compression therapy and contributes to the treatment of chronic venous insufficiency.

## Introduction

Compression therapy in chronic venous insufficiency (CVI) must be feasible and sustainable to maximize the therapeutic effects. However, in many cases, treatment is discontinued owing to physical and/or psychological problems such as orthopedic disorders, obesity, dementia, lack of understanding, iatrogenic skin disorders, financial burden, and lack of support systems, especially in older adults. To improve adherence, some healthcare providers prefer the use of single or superimposed hosiery with low compression pressure^1)^ or larger socks.^2)^ Attempts to provide support through visiting nurses could increase the patients’ financial burden.

In this context, adjustable compression garments (ACGs) are useful for improving adherence to compression therapy.^3^^,^^4)^ Some reports have stated that ACGs have the advantage of being self-adjustable,^5)^ have a reliable compression pressure for self-adaptation,^6)^ and have a therapeutic effect superior to inelastic bandages in ulcer cases.^7)^ However, in older adults aged >65 years, the limiting factors for applying ACGs are not age issues but obesity, decline in cognitive function, weak grip strength, and low social status.^8)^ Therefore, the ease of use and availability of ACGs must be improved to encourage their use.

In contrast, compression by a non-stretchable inelastic material, which exerts low resting and high working pressures, is well tolerated by patients and is more effective in reducing edema, enhancing muscle pump action, and increasing arterial inflow than low-stretching elastic materials.^9^^–^^11)^

Therefore, we developed a new ACG, McBoooon (Mc), manufactured using an inelastic fabric. When the straps are attached to the body of the garment, the outer and back parts of the fabric are firmly secured without hook-and-loop fasteners or metal hooks, and minimal force is required to detach the straps. This study reports the results of an assessment of its physical features, a comparative study of its compression properties in vivo, and its effect on the ejection peak velocity (PV) of the popliteal vein compared with an ASHIKA stocking (AS; Integral Co. Ltd, Tokyo, Japan), which is a widely used medical elastic knee-high compression stocking in Japan. According to the manufacturer, the compression pressure exerted by an AS at the ankle is 20–26 mmHg.^12)^

### A new ACG , Mc

**Figure 1:**
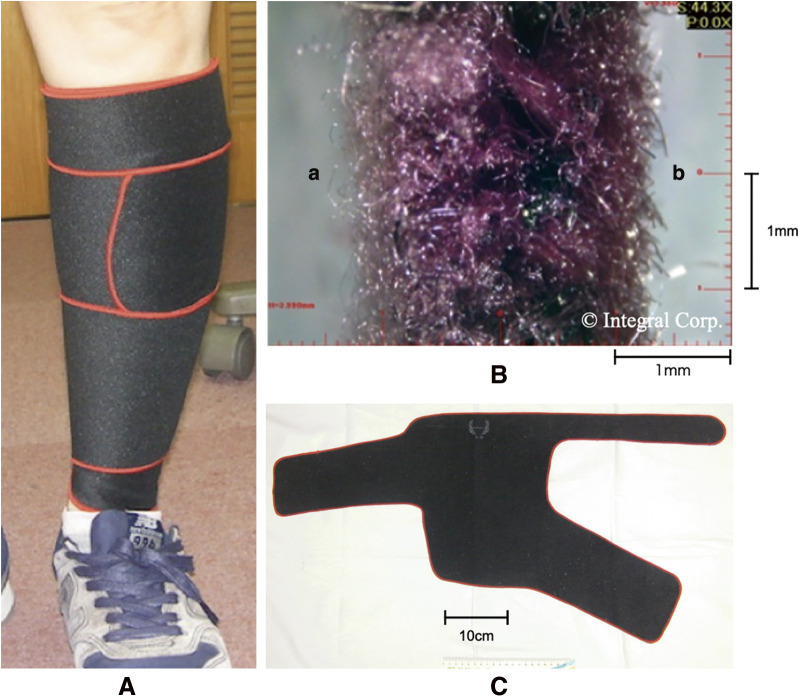
Fig. 1 The McBoooon. (**A**) Front view of applied McBoooon. (**B**) Magnified cross-section of fabric, a: Loop face, and b: hook face. (**C**) Geometry net of McBoooon.

The Mc, which is made of 100% polyester, consists of a main body and three straps. The loop face (side A) and the hook face (side B) adhere to each other. The proximal strap (S1) is positioned slightly below the knee, the middle strap (S2) is wrapped around the calf at its maximum diameter (C), and the distal strap (S3) is firmly wrapped around the level of the medial gastrocnemius muscle transition into the Achilles tendon (B1) and ankle (**Fig. 1**). The weight of the entire garment is 57 g for size S–M and 78 g for size L–XL.

**Figure 2:**
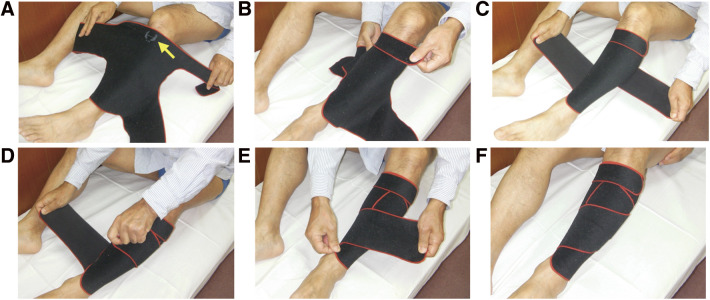
Fig. 2 Process of applying McBoooon (left leg). (**A**) The Mc is placed over the lower leg just below the knee using the logo (yellow arrow) as a guide. (**B**) S1 is wrapped backward from the outside of the leg and attached to the main body. (**C**) S2 and S3 are pulled in opposite directions with equal force. (**D**) S2 is wrapped around C. (**E**) S3 is wrapped tightly from the ankle to B1. (**F**) Completion to wrap around the right lower leg, the Mc is turned over and used in a similar manner.

The Mc should be wrapped around the limb in the sitting position while stretching out the leg because the circumference of the leg increases when bearing weight. To wrap the left lower leg, the Mc is placed over the lower leg using the logo as a guide. First, S1 is wrapped backward from the outside of the leg and attached to the main body of the garment. Next, S2 and S3 are pulled in opposing directions with equal force. Then, S2 is wrapped around C, and S3 is wrapped tightly around B1 to prevent slippage (**Fig. 2**). To apply the Mc to the right lower leg, the garment can be turned over and applied similarly.

The physical features of the fabric were evaluated by a third-party national organization (BOKEN Quality Evaluation Institute, Tokyo, Japan). According to their report, the stretching rate of the fabric measured using the JIS-L-1096-D method is 4.2% and the tensile shear strength and peel strength while sides A and B are attached measured using the JIS-L-3416 method are 1.1 N/cm and 0.14 N/cm, respectively. Air permeability measured using the JIS-L-1096-A method is 36.1 cm/cm/s, while that of AS measured using the same method is 30.6 cm/cm/s.

## Materials and Methods

**Figure 3:**
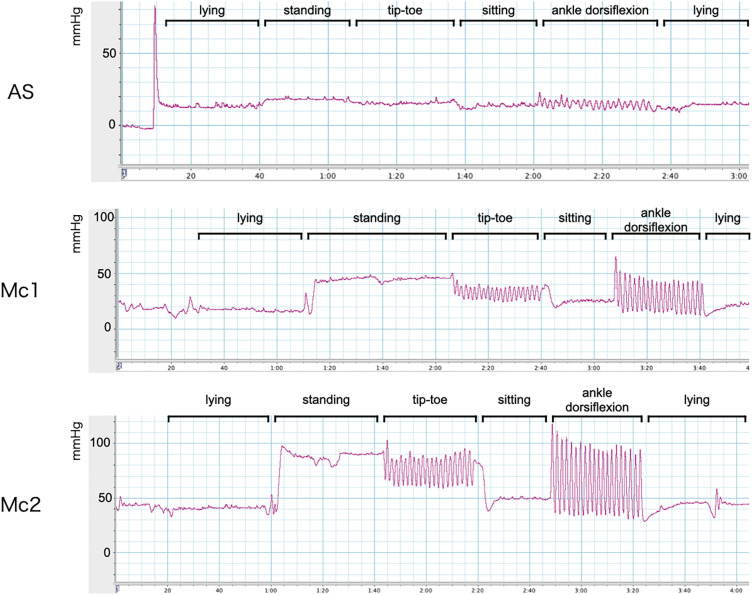
Fig. 3 Representative graph of the interface pressure. AS: ASHIKA stockings; Mc1: McBoooon wrapped with the same initial interface pressure as that of AS; Mc2: McBoooon wrapped with approximately twice the initial interface pressure of Mc1.

Twelve healthy volunteers (6 males and 6 females, mean age of 47.6 years [range, 36–64 years], mean circumference at the ankle (B) of 20.3 cm [range, 17.7–21.8 cm]) were investigated. The interface pressure (IP) was continuously measured at B1 during body changes and movements. The participants were asked to rest in the supine position with an air-sensing cuff attached to the left lower limb at B1 for pressure measurements. After applying AS to the limb, IP measurements were initiated. Participants were asked to remain standing until the IP stabilized, perform the tiptoe exercise 20 times, remain sitting until the IP stabilized, perform ankle dorsiflexion 20 times, and finally return to the supine position. Then, the IP was measured as described above, wrapping the Mc with the same initial resting IP as that of AS (Mc1) and with an IP approximately twice that of AS (Mc2). **Figure 3** shows a representative graph of the IP in the three groups. The size of the AS was selected based on the participant’s circumference at B according to the manufacturer’s recommendations.

The IP measuring device was an A0905-SA-SP manufactured by AMI-Techno, Tokyo, Japan. The measured signal was recorded by the RMT-1000 polygraph system (Nihon Kohden, Tokyo, Japan) and analyzed using LabChart version 8 (ADInstruments; Bella Vista, NSW, Australia). The IP sampling rate was set to 20 samples/s.

To calculate the static stiffness index (SSI), defined as the difference in IP between the supine and standing positions, the IP values in the most stable one-second interval (20 samples) for each posture were extracted. The SSI was calculated as the average of the extracted values. The dynamic stiffness index (DSI) during the tiptoe exercise (DSI-tip) and ankle dorsiflexion (DSI-dor) was calculated as the average of the 9th–18th amplitudes extracted from 20 exercises.

On a different day, the PV was evaluated in ten participants using ultrasonography (Hitachi HI VISION Avius; Hitachi, Tokyo, Japan). With the participant sitting, a linear probe (7.5 MHz) was applied to the popliteal fossa, and the flow velocity waveforms associated with 20 ankle dorsiflexion movements were continuously recorded. The PV was calculated as the average PV in 10 samples from the 9th–18th dorsiflexion exercises, and the values for no compression, AS, Mc1, and Mc2 were compared.

Data were entered into Microsoft Excel, and the statistical program file ystat2013 (Shinya Yamazaki, Igakutosho Shuppan, Tokyo, Japan) was used for analysis. The Wilcoxon t-test was used to compare differences. Unless otherwise specified, the results were expressed as the median [range], and p <0.05 was considered significant.

This study was approved by the Medical Association Ethics Committee of Kagawa Prefecture (approval number: 2022-5).

## Results

The initial resting IP values (mmHg) for AS, Mc1, and Mc2 were 20.8 [13.2–26.7], 20.5 [12.7–28.5], and 40.7 [34.2–48.2], respectively. Although no statistically significant difference was found between IP values in AS and Mc1 (p = 0.942), a significantly higher IP was observed in Mc2 (p = 0.002 in each group).

The SSI values (mmHg) were 3.1 [–2.4–7.7] for AS, 26.9 [16.4–41.8] for Mc1, and 46.8 [19.0–82.5] for Mc2, with significant differences (p = 0.002 for AS vs Mc1 and Mc2, p = 0.006 for Mc1 vs Mc2). The DSI-tip values (mmHg) were 2.5 [1.4–6.1] in AS, 9.8 [3.2–17.8] in Mc1, and 19.6 [7.0–43.1] in Mc2, with significant differences (p = 0.002 in each group). Furthermore, the DSI-dor values (mmHg) were 4.6 [2.4–8.2] in AS, 18.6 [7.3–29.2] in Mc1, and 37.3 [17.9–62.8] in Mc2, with significant differences (p = 0.002 in each group). The DSI-dor values were approximately twice as high as the corresponding DSI-tip values in each group.

**Figure 4:**
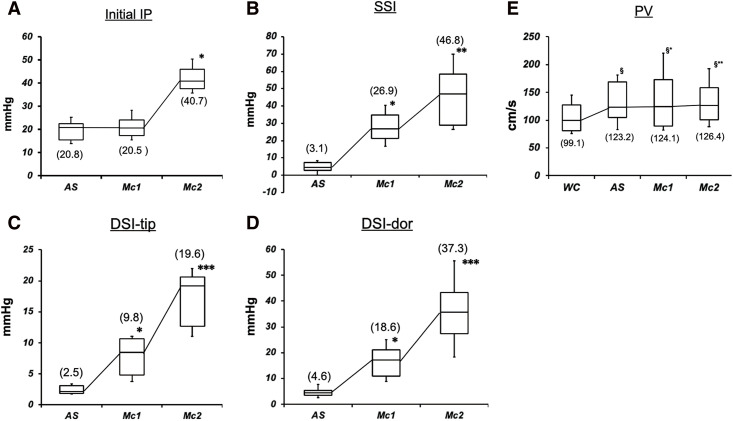
Fig. 4 Comparison of stiffness and peak ejection flow velocity. (**A**) IP: interface pressure. (**B**) SSI: Static stiffness index. (**C**) DSI-tip: Dynamic stiffness index with tiptoe movement. (**D**) DSI-dor: Dynamic stiffness index with ankle dorsiflexion. (**E**) PV: Peak velocity. AS, ASHIKA stockings; Mc1: McBoooon wrapped with the same initial interface pressure as that of AS; Mc2: McBoooon wrapped with approximately twice the initial interface pressure of Mc1; WC: without compression (median). *p = 0.002 for AS vs Mc1 **p = 0.006 for Mc1 vs Mc2 ***p=0.002 for Mc1 vs Mc2 §p = 0.017 vs WC §*p = 0.047 vs WC §**p = 0.007 vs WC

The PV values (cm/s) were 99.1 [69.3–148.8] in the control measurements without compression (WC), 123.2 [64.4–214.4] in AS, 124.1 [84.7–219.2] in Mc1, and 126.4 [82.1–226.2] in Mc2. Significantly higher PV values were detected in AS, Mc1, and Mc2 compared with WC (p = 0.017 for WC vs AS, 0.047 for WC vs Mc1, 0.007 for WC vs Mc2); however, there were no significant differences between the three groups (p = 0.887 for AS vs Mc1, 0.335 for AS vs Mc2, and 0.736 for Mc1 vs Mc2) (**Fig. 4**).

The findings of this study can be summarized as follows:

1. Mc was classified as an inelastic ACG.
2. The SSI was approximately 8 times higher in Mc1 and 15 times higher in Mc2 than in AS.
3. Both DSI-tip and DSI-dor in Mc increased as the initial IP increased.
4. DSI-dor was approximately 2 times higher than DSI-tip in every group.
5. Although PV significantly increased with any compression, no significant differences were found between the 3 groups.

## Discussion

Regarding compression therapy for CVI, a consensus has been reached based on clinical class in the Clinical-Etiological-Anatomical-Pathophysiological (CEAP) classification^13)^ and applied pressure at the ankle level; the compression pressure at ankle level ≥15 mmHg should be applied for patients with only venous symptoms, 20–40 mmHg for patients with C3 and C4, and ≥40 mmHg for patients with C6.^14)^ Graduated pressure from the ankle upwards is recommended, but some reports have stated that applying more pressure to the calf than to the ankle may enhance the muscle pumping action^15)^ or that reversed graduated pressure is more likely to improve occupational edema.^16)^ The optimal method for compression remains undetermined.

However, reports have suggested that the high stiffness of inelastic materials effectively reduces swelling and promotes ulcer healing. Several ACGs have been developed to facilitate the use of inelastic materials. Based on several sources, the Clinical Practice Guidelines of the European Society for Vascular Surgery published in 2022 recommend that these ACGs should only be applied in C5, C6, and certain C3 cases.^13^^,^^14)^ In the guidelines, specific compression pressure values are recommended for pathological condition; however, although higher stiffness is required as the clinical classification progresses, the values are not specified. Furthermore, it remains unclear whether compression pressure or stiffness should be prioritized during compression therapy for CVI.

Current ACG designs utilize hook-and-loop or metal hook fasteners. In addition, the attached area is limited to the area of the hook-and-loop fastener, which requires strong adhesion to resist tension and pulling force. The fabric of the main body is easily damaged. In contrast, the Mc has stronger resistance to tension because the entire areas of sides A and B are securely attached. Although no official information is available on the peeling strength of Velcro wrap devices currently on the market, the Mc requires less force to detach the strap from the body than to pull it off.

Benigni et al. reported the stretch rates, compression pressures, and SSIs of six different ACG products.^17)^ Except for Coolflex, all these products (Juzo Compression Wrap 6000, ReadyWrap, JuxtaFit, JuxtaLite, and CompreFlex) are made of low-stretch or high-stretch materials. In the latter case, the degree of compression can be adjusted by adjusting the stretching rate of the strap. When a resting IP of approximately 20 mmHg was applied, the SSI was 8–11 mmHg. However, because Coolflex consists of non-stretch material, exerting >30 mmHg at rest was not achievable; when the average resting IP was 19 mmHg, the average SSI was 16 mmHg. In our study, the Mc exerted a maximum resting IP of 48.2 mmHg, and when the resting IP was approximately 20 mmHg, the median SSI was 26.9 mmHg. These results indicate that the Mc can exert higher IP and SSI than Coolflex.

This may be attributable to the difference in the material composition. According to the manufacturer’s information, Coolflex is composed of 47% polyester, 25% polyamide, 20% cotton, 7% elastane, and 1% stainless steel, whereas Mc is composed of 100% polyester.

However, because the Mc is non-stretchable, the compression pressure cannot be adjusted by the stretching rate of the strap, and the exact compression pressure cannot be determined unless the IP is measured. For clinical applications, we first used a compression pressure of approximately 20 mmHg at B1, and then continued to increase the pressure. If S2 and S3 are wrapped equally tightly, a pressure gradient is obtained according to Laplace’s law. However, the compression pressure gradient between these straps is affected by the difference in the circumferences of C and B1. In addition, the higher the resting pressure of B1, the higher the SSI and DSI values. As long as it was tolerable, we instructed the patients to wrap the Mc as tightly as possible without causing pain.

The Mc was designed primarily to provide a high degree of stiffness. Below B1, no intramuscular veins are present; therefore, not covering the ankle is unlikely to affect muscle pump function. However, in cases where ulcers are found below the distal leg, we recommend applying the Mc in combination with ulcer compression using an elastic or inelastic bandage. We could not find any reports on the relationship between the DSI and venous hemodynamics. The DSI has a strong positive correlation with the SSI.^18)^ Because most patients move their legs to some degree daily, the DSI is expected to have important implications. In this study, the DSI was approximately twice as high in dorsiflexion than in plantar flexion regardless of the compression device used. Suehiro et al. demonstrated that the refilling volume (ejection volume) was larger during dorsiflexion than during the tiptoe exercise, as assessed by air plethysmography, and that ankle dorsiflexion plays a more important role than tiptoe in venous return from the foot and lower leg.^19)^ The contraction and relaxation patterns of the lower leg muscles during ankle dorsiflexion in the sitting position are similar to those during the swing phase of walking. In this phase, the gastrocnemius venous sinus is empty and under negative pressure, functioning not as a reservoir but as a conduit from the network of intramuscular veins to the axial deep veins.^20)^ The high counter pressure exerted by the Mc should augment venous flow from the intramuscular to the axial deep veins.

Partsch et al. described counterpressure during ambulation as a massage effect.^21)^ A high DSI provides a large pressure gradient in the deep veins owing to the rapid rise and fall in the IP. Because the blood flow velocity depends on the pressure gradient, a rapid decrease in the IP leads to augmented venous flow from the superficial to the deep region and from the sole to the leg. Shear stress on the endothelium of the deep veins also increases.

**Figure 5:**
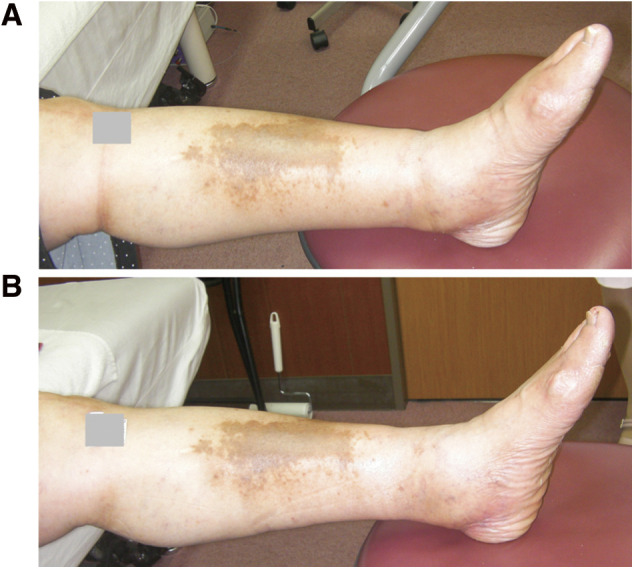
Fig. 5 Edema reduction by McBoooon (67-year-old female). (**A**) Lower leg and foot edema remained after wearing AS for 1 year. (**B**) Edema markedly reduced by applying the Mc for 10 days.

**Figure 5** shows a 67-year-old woman who underwent endovenous laser ablation for superficial varicose veins. One year after AS administration, chronic edema persisted (**Fig. 5A**). Then, after ten days of application of the Mc with the same IP as AS, edema in the lower leg and foot disappeared, even after foot-sparing compression (**Fig. 5B**). This phenomenon suggests that higher stiffness can contribute to reducing venous hypertension and improving microcirculation more than the IP.

The significantly higher PV in every compression group compared with that in the no-compression group was assumed to be related to the reduced diameter of the deep vein. Although several studies on patients with CVI have shown that compression therapy enhances muscle pump function assessed by ejection volume and fraction using plethysmography,^5)^ in this study, a small number of healthy volunteers with no significant stenosis or regurgitation at the sampling point were included, which is why no significant difference was found among the three groups.

We are currently applying the Mc in cases C0s–C6, including those with low adherence.

We plan to report on its therapeutic effects in the near future.

## Conclusion

The physical features and compression characteristics of a newly developed ACG are described in this report. The Mc is classified as a non-stretch inelastic garment designed to increase stiffness and improve adherence to compression therapy. Although we have had few clinical applications, the higher stiffness of the Mc compared to that of the AS augments venous return and contributes to reducing leg edema in patients with CVI. We hope that this report will contribute to the treatment of CVI, considering that the Mc can be used alone or in combination with other compression materials, depending on the pathological condition.

## Acknowledgments

The authors thank Integral Co., Ltd. and Kagawa Seamless Co., Ltd. for their efforts in commercializing the McBooon.

## Disclosure Statement

The authors declare no conflict of interest.

## Additional Remarks

The contents of this paper were presented at the 43rd Annual Meeting of the Japanese Society of Phlebology held in Matsuyama City, Japan, in 2023.

## Author Contributions

Study conception: YS

Data collection: AM, KI, NK, and MO

Analysis: YS

Investigation: YS

Manuscript preparation: YS

Critical review and revision: all authors

Final approval of the article: all authors

Accountability for all aspects of the work: all authors
